# Animal Models for the Genetic Study of Human Alcohol Phenotypes

**Published:** 2002

**Authors:** Tamara Phillips

**Affiliations:** Tamara Phillips, Ph.D., is a professor at Oregon Health & Science University and a research career scientist at the Veterans Affairs Medical Center, Portland, Oregon

**Keywords:** genetic theory of AODU (alcohol and other drug use), alcoholic beverage, phenotype, animal model, gene knockout, transgenic technology, QTL (quantitative trait locus) mapping, mutagenesis, gene expression, genetic trait, genetic correlation analysis

## Abstract

Researchers are increasingly using animal models to study the genetic basis of complex human behaviors, such as alcoholism. The most commonly used animal species are rodents, but other species, such as nonhuman primates, fruit flies, and zebrafish, can also provide important information. A variety of approaches are employed in these studies, particularly knockout and transgenic mice as well as specially bred animal lines that can be used for various genetic analyses, including quantitative trait locus mapping. Other strategies applied in genetic studies in animal models include random mutagenesis, virus-mediated gene transfer, and gene expression profiling.

Vertebrate and invertebrate animal models contribute to researchers’ knowledge about the genetics of alcoholism. Overall, the DNA sequences of humans and other organisms (e.g., other primates, rodents, and even fruit flies or yeast) are rather similar (e.g., Bihoreau et al. 2001). Therefore, findings regarding the association of certain genes with specific alcohol-related physiological changes and behaviors (i.e., phenotypes) in animals can at least to some degree be extended to human alcoholics and vice versa. In fact, some researchers have suggested that mouse lines carrying human genes—an approach called gene-swap—will become a common method for studying both the effects of specific genes and genetic differences among humans (Nebert et al. 2000).

One example of how genetic discovery in human alcoholics can be translated to animal research involves the dopamine D_2_ receptor gene (called *DRD*_2_ in humans and *Drd*_2_ in mice). Dopamine is a brain chemical (i.e., neurotransmitter) used in the communication between nerve cells. To exert its effects, dopamine is released from the signal-emitting cell and binds to docking molecules (i.e., receptors) on the surface of the signal-receiving cell. Several genes encode dopamine receptors, including *DRD*_2_. More than a decade ago, some studies implicated variations in the *DRD*_2_ gene as a factor influencing a person’s risk of developing severe alcoholism, although subsequent studies have provided mixed support for this claim (e.g., Noble 2000; Matsushita et al. 2001). Subsequently, studies using a technique called quantitative trait locus (QTL) mapping (described in more detail in the section “Specific Genetic Approaches”) indicated that a region on mouse chromosome 9, which is similar to a region on human chromosome 11 where the *DRD*_2_ gene resides, influenced voluntary alcohol consumption in mice (Phillips et al. 1994). Together with the clinical results obtained in humans, this finding led researchers to consider the mouse *Drd*_2_ gene as a good candidate for further study. Followup work in mice that lacked functional D_2_ receptors (knockout mice, which are also described in the section “Specific Genetic Approaches”) provided further evidence of the involvement of this receptor because the knockout mice exhibited markedly reduced alcohol consumption compared with normal littermates (Phillips et al. 1998).

Researchers also introduced a modified virus into a specific brain region of rats called the nucleus accumbens, which plays an important role in drug reward. The modified virus produced excessive amounts of the D_2_ receptor and resulted in reduced alcohol consumption and preference in the animals (Thanos et al. 2001).^1^ Although these findings do not prove that the gene identified by QTL mapping on mouse chromosome 9 is *Drd*_2_, they do provide justification for continued studies of the role of this gene and its product in alcohol consumption.

This article describes several of the genetic methods that have been used in various animal models of alcohol research, including rodents and other species. It is important to realize, however, that for such complex diseases as alcoholism, animal models cannot *prove* genetic associations in humans. Human alcoholism cannot be exactly replicated in animals, even though some external factors thought to influence human alcohol consumption, such as physiological and social stress, can be modeled in animals.

Despite these limitations, the ability to precisely control environmental and genetic characteristics of animal models allows researchers to identify important avenues of investigation that can then be translated to research in humans. For example, because the gene frequencies can be changed in animals by selective mating or by specific mutations introduced through genetic engineering, one can systematically study changes in the influence of a single gene that are caused by this gene’s interactions with other genes (i.e., the genetic background). Ultimately, careful investigation of gene–gene interactions in human populations will contribute to a more complete understanding of the coordinated biological systems that influence the susceptibility to and progression of alcoholism as well as the effectiveness of alcoholism treatment.

## Specific Genetic Approaches

### Knockout Mice and Transgenic Mice

When a known gene is suspected to influence a trait (e.g., sensitivity to alcohol), researchers can disrupt or enhance the function of that gene using molecular genetic techniques (Homanics 2002) and then study the effects of this genetic alteration. For example, mice in which the function of a particular gene has been disrupted—called knockout mice—are increasingly being used in research (Anagnostopoulos et al. 2001), including numerous studies in alcohol research. The disruption of gene function also has been applied to more “primitive” organisms, such as yeast, to study alcohol as a stressor (i.e., a stimulus that activates certain “stress-related” signaling pathways in the organism) (Zu et al. 2001). Furthermore, disruption of gene function is a classic approach in genetic analyses of the fruit fly *Drosophila melanogaster* (discussed in the section “Research in Other Species”). Similarly, researchers are examining the functions of a particular gene by introducing that gene into other organisms, generating “transgenic” animals (most commonly mice).

Knockout and transgenic mice have been used in various areas of alcohol research, such as studies of the genetic basis of alcohol’s pleasurable (i.e., rewarding) effects (for a review, see Cunningham and Phillips 2003). In many cases, a particular transgenic or knockout animal is studied because pharmacological or electrophysiological evidence implicated the gene product that is altered in that animal. Evidence from human linkage studies—studies in which researchers analyze whether people with a certain trait (e.g., alcoholism) are more likely to carry a particular variant of a gene or DNA region—probably has also influenced research in these animal models (e.g., Phillips et al. 1998; Deltour et al. 1999).

Certain difficulties, however, are associated with interpretation of data generated using transgenic or knockout animals (e.g., Gingrich and Hen 2000). In particular, any interpretation must take into consideration the difference between variation in gene function associated with different variants (i.e., alleles) of a gene versus the obliteration of function as a consequence of gene knockout technology. In some instances, a disease develops when the function of a specific gene is completely absent. In other cases, variation in disease susceptibility appears to be associated with altered, rather than absent, gene function. In these instances, when elimination of gene function affects a trait (e.g., alcohol consumption), one cannot conclude that this gene is associated with the trait variation (i.e., differences in alcohol consumption levels) seen in “natural” populations.

### Quantitative Trait Locus (QTL) Mapping

Quantitative traits are genetically influenced characteristics that differ in the extent to which each individual in a population possesses that characteristic (e.g., height or sensitivity to alcohol), rather than in the kind of trait they possess (e.g., eye color). Quantitative traits are generally determined by more than one gene, each of which can exist in several forms (i.e., alleles), as well as by multiple environmental factors. Thus, differences in the trait among the individuals in a population most commonly result from variations in gene function rather than from the presence versus absence of gene function. The DNA regions that influence quantitative traits (i.e., that contain genes determining the trait) are called QTLs. QTL mapping takes advantage of naturally occurring genetic variation to determine the regions of specific chromosomes that harbor genes influencing quantitative traits.

For QTL mapping analyses, researchers can use different types of genetically defined animal populations, including the following (Palmer and Phillips 2002):

- *Recombinant inbred (RI) strains*. These strains are generated by repeatedly inbreeding brother–sister pairs from the second-generation (F_2_) offspring of two genetically distinct parent inbred strains. Because of genetic recombination of the DNA from the parent strains that occurs during the production of sperm and egg cells, each F_2_ animal has a slightly different combination of genes from the parent strains. By repeated inbreeding, animal strains are generated in which all the animals within a strain are genetically identical, but each strain differs in the specific combination of parental genes. (For more information on this approach, also see the article by Dick and Foroud, pp. 172–180.).
- *Chromosome substitution strains (CSS)*. To create a CSS, an entire chromosome of one inbred strain is replaced with that of another strain. The final product is a mouse of one strain that has been altered only with regard to replacement of a single pair of chromosomes from another “donor” strain.
- *Intercross and backcross populations*. An intercross population is the F_2_ offspring of two genetically distinct inbred strains. A backcross population is produced by mating two genetically distinct inbred strains to produce the first generation (F_1_) and then breeding the F_1_ offspring back to one of the parent inbred strains.
- *Selected lines*. These lines are discussed in the next section.
- *Heterogeneous populations*. Individuals within these populations have diverse genetic backgrounds. The most commonly used heterogeneous mouse stock was produced by interbreeding eight genetically diverse inbred strains.

Finer mapping of a QTL to the smallest possible genetic segment may involve the use of specialized genetic models (e.g., models called interval-specific congenic strains [ISCS] and advanced intercross lines [AIL]) as well as of heterogeneous populations (see Palmer and Phillips 2002).

Alcohol-related traits have been most extensively mapped in mice (see Crabbe et al. 1999). To identify specific genetic variants associated with a particular alcohol effect, it is important to narrow the DNA region possessing a QTL down to one that is likely to contain only a few genes (see Palmer and Phillips 2002). One trait for which researchers have made considerable progress is relative sensitivity to alcohol’s sedative effects (Ehringer et al. 2001). Any genes that are identified in such a mouse model of alcohol sensitivity may prove important for investigations in human populations, because relative insensitivity to alcohol may be related to increased alcoholism risk (e.g., Heath et al. 2001).

### Selected Lines

Selected lines are generated by selectively breeding animals within a population that have either very high or very low levels of the desired trait (e.g., alcohol consumption). When the selective breeding is conducted over several generations, the selected lines often show progressively greater differences from each other with respect to the trait under investigation and with respect to the gene variants that influence the trait. The lines should remain similar, however, with respect to traits not influenced by those genes. Selective breeding approaches have resulted in several rat and mouse lines that have extremely high or low sensitivity, preference, or aversion to alcohol. Some of these lines have also been shown to exhibit different types of adaptation in brain function (i.e., neuroadaptation) in response to alcohol exposure. Selected lines are frequently used to identify traits that are influenced by common underlying genetic factors.

Selected lines also are being used in QTL mapping studies to locate the genes that influence the selected trait. For example, a study comparing rats that were selectively bred to consume either large amounts of alcohol (i.e., alcohol-preferring, or P, rats) or small amounts of alcohol (i.e., alcohol-nonpreferring, or NP, rats) identified a QTL near the neuropeptide Y (*NPY*) gene (Carr et al. 1998). NPY is a small protein molecule (i.e., a peptide) that has been suggested to have diverse functions, including the control of food intake, emotional behavior, and responses to alcohol. Studies in other genetic models (e.g., knockout mice) and neurochemical studies in selected lines have supported a role for NPY in alcohol consumption (Tecott and Heberlein 1998). The results regarding the function of NPY are equivocal, however, because no QTL in that gene region was found in a different set of high-alcohol-drinking (HAD) and low-alcohol-drinking (LAD) rat lines (Foroud et al. 2000). These contradictory findings may result from differences in the genetic backgrounds of the rats used in the two studies.

Similarly discrepant results regarding the role of NPY have been found in humans. Thus, one study in humans found that a certain *NPY* gene variant was associated with increased alcohol consumption (Kauhanen et al. 2000), whereas in a subsequent investigation this gene variant was found less frequently among alcoholics (Ilveskoski et al. 2001). This discrepancy may result from differences in the phenotypes that were examined in the two studies. Future research needs to clarify the exact role of the *NPY* gene in influencing alcohol consumption and alcoholism risk as well as the exact sources of the discrepancies in the existing findings.

### Random Mutagenesis

Researchers use transgenic or knockout mice to study the specific effects of a known gene. To identify new genes that might affect a trait of interest, however, they may use an approach called random mutagenesis. With this approach, the animals (e.g., mice) are treated with a chemical that can induce changes (i.e., mutations) in the DNA of the sperm or egg cells so that the mutations are passed on to the offspring of those animals. Consequently, every gene becomes a potential target for study. In 1999, seven institutes of the National Institutes of Health (NIH), including the National Institute on Alcohol Abuse and Alcoholism (NIAAA), initiated a mouse genetics research program to launch genomewide mutagenesis projects in mice and develop methods allowing rapid screening of large numbers of phenotypes resulting from such random mutagenesis (Moldin et al. 2001). The hope is that these genetic tools will lead to broad insights in the study of complex behaviors, such as alcohol consumption. However, researchers will be facing significant hurdles in achieving this goal. For example, phenotypic screening of animals with such mutations is most likely to detect major gene effects and may miss more subtle effects. Furthermore, the identification of specific mutation(s) associated with a complex trait will require extensive mapping like that used in QTL analyses (Belknap et al. 2001). The fruits of this approach in addiction research await harvesting.

### Virus-Mediated Gene Transfer

A compelling approach to confirming the involvement of a specific gene in a trait thought to be controlled by a specific region of the central nervous system is to transfer that gene into that specific region and then look for changes in the trait. One way to transfer the gene is to insert it into a modified virus that can then be used to infect a specific cell type but is not harmful to the animal or human being studied. Once the virus infects its target cells, it begins to produce the product of the inserted gene, resulting in abnormal or higher-than-normal levels (i.e., overexpression) of that gene product in the infected cells. The technology associated with this virus-mediated gene transfer is improving (Carlezon et al. 2000), and the method is being used in alcohol research. For example, as mentioned previously, researchers have demonstrated an association of *Drd_2_* overexpression with reduced alcohol consumption using virus-mediated gene transfer (Thanos et al. 2001). Other investigators are studying the role of certain enzymes in alcohol-induced liver injury using virus-mediated gene transfer of those enzymes (Wheeler et al. 2001). This approach may have far-reaching implications for the prevention and treatment of alcoholism and other addictions.

### Gene Expression Profiling

Researchers are also conducting large-scale studies of changes in gene expression (i.e., in the amount of gene product produced) that may be associated with alcoholism and other addictions (Uhl et al. 2001). Using technologies called gene chip or microarray analyses, these investigators examine the expression of thousands of genes simultaneously from a single DNA sample. This approach is also called gene expression profiling. It allows researchers to collect information about genetic changes that occur together in a coordinated fashion to affect a given phenotype, rather than being limited to the examination of one gene at a time. For example, alcoholics often exhibit loss of white matter in various brain regions. This white matter is composed of the extensions of nerve cells that are normally covered by an insulating substance called myelin. Using gene expression profiling, researchers were able to demonstrate reduced expression of several myelin-related genes in the brain tissue of alcoholics obtained after they died (Lewohl et al. 2001).

Animal studies already have identified many genes whose expression is altered after alcohol and other drug administration; moreover, such analyses demonstrated that different genetic changes are associated with alcohol’s effects after initial and long-term (i.e., chronic) consumption. One example of animals that have been profiled for differences in gene expression are mice that were selectively bred for extreme differences in sensitivity to alcohol’s sedative (i.e., hypnotic) effects (Xu et al. 2001). In other work, a gene called cocaine- and amphetamine-regulated transcript (*CART*) was found to change its expression in response to cocaine and amphetamines. This gene was further studied by injecting a fragment of the *CART* gene product into the ventral tegmental area of rats, a brain region thought to influence the experience of drug reward. Injection of this fragment induced behaviors virtually identical to those seen after cocaine administration (Kimmel et al. 2000). Similar work is being conducted to study other genes whose expression is found to be changed in response to alcohol.

## Research in Other Species

Researchers also are studying the genetics of alcoholism and other addictions in species other than rodents and humans. For example, changes in gene expression in response to chronic cocaine treatment have been described in certain brain areas of nonhuman primates (Freeman et al. 2001), and similar analyses are likely to be performed in nonhuman primate models of alcoholism.

Another commonly used animal model is the fruit fly *Drosophila melanogaster*, which has a long and rich history in genetic research. The entire *Drosophila* DNA sequence has been deciphered and has shown considerable similarity (i.e., homology) with that of mammals (Adams et al. 2000). Several laboratories have studied *Drosophila* genes that influence sensitivity or tolerance to alcohol (see Heberlein 2000). One *Drosophila* mutant with markedly increased alcohol sensitivity has been named “cheap-date” (see Heberlein 2000). The mutation in this strain affects a signaling system called the cAMP pathway, which is important for many regulatory processes in the cell. The cAMP signaling pathway also has been implicated in determining alcohol consumption and sensitivity to alcohol in studies of a mouse knockout model (Thiele et al. 2000) and of human alcoholics (Yamamoto et al. 2001). These observations suggest that *Drosophila* will provide an important model system for at least some aspects of alcohol’s effects.

Studies in various animal models sometimes also complement or inform each other. For example, the findings regarding the role of the cAMP signaling pathway just described are supported by studies in the round worm (i.e., nematode) *Caenorhabditis elegans*. Analyses in that organism identified a gene that is also part of the cAMP pathway and which influences adaptation to another addictive substance, nicotine (Waggoner et al. 2000). Similarly, non-alcohol-related research in *Drosophila* has led to the isolation of a mouse gene that corresponds to a *Drosophila* gene called *neuralized*, which subsequently was found to alter sensitivity to alcohol (Ruan et al. 2001).

Another organism that is increasingly used in research on alcoholism and other addictions is the zebrafish *Danio rerio*. The embryos of these fish are transparent, allowing easy anatomical characterization that facilitates genetic analysis. Furthermore, for many zebrafish genes, corresponding genes exist in mammals. For example, researchers have cloned a zebrafish gene that corresponds to a mammalian receptor for opioid drugs (e.g., morphine) and have studied that receptor’s distribution in the zebrafish’s central nervous system (Porteros et al. 1999). This research may have implications for alcohol research in humans because drugs that interfere with the activity of opiates and their receptors (i.e., opiate antagonists) have shown promise in alcoholism treatment (Kranzler and Van Kirk 2001). Researchers also have recently cloned the zebrafish gene for alcohol dehydrogenase (ADH), the primary enzyme responsible for the degradation of alcohol in the body (Dasmahapatra et al. 2001). This gene also exhibits substantial genetic similarity to a human *ADH* gene. Finally, zebrafish exhibit behavioral responses to alcohol that are reminiscent of those seen in other model organisms (Gerlai et al. 2000). Consequently, research using zebrafish may be a promising approach for identifying important genetic influences on alcoholism.

## Concluding Remarks

The search for genes related to alcoholism using such methods as QTL mapping and random mutagenesis is a painstaking process because of the difficulty of sifting through large amounts of genetic information to identify relevant genes. Additional genetic approaches (e.g., comparative sequence analyses) are being used to identify differences in the genetic material within genetic regions implicated by QTL analyses that could be functionally relevant to the trait associated with a given QTL. However, finer mapping techniques are still needed to narrow down long lists of alleles potentially related to the trait under investigation. Investigators are also trying to integrate gene profiling information from animals with results from human linkage studies in order to identify high-probability candidate genes for further analyses. However, such “one-gene-at-a-time” approaches are beginning to give way to synthetic approaches that consider interacting genes and coordinated pathways that influence complex traits. The wealth of sequencing information now available in various species, the development of high-throughput methods for genetic analyses, and the evolution of methods for handling massive data sets should all accelerate the identification of genes that influence alcohol’s effects and the risk of alcoholism.

## Footnotes

1 One would expect that if mice which lack a functional D_2_ receptor exhibit reduced alcohol consumption, animals producing excessive amounts of that receptor should exhibit increased alcohol consumption. The prediction is not that straightforward, however. The knockout mice, which lack the receptor, likely no longer experience the rewarding effects of alcohol that are modulated by that receptor; accordingly, their alcohol self-administration drops. The animals with the excessive receptor levels, in contrast, receive a stronger rewarding effect after consuming alcohol. As a result, they will consume less alcohol to get the same rewarding effect as rats with normal receptor levels.
